# Recent Advances in Luminescence Imaging of Biological Systems Using Lanthanide(III) Luminescent Complexes

**DOI:** 10.3390/molecules25092089

**Published:** 2020-04-29

**Authors:** Jorge H. S. K. Monteiro

**Affiliations:** Department of Chemistry, Humboldt State University, Arcata, CA 95521, USA; jorge.monteiro@humboldt.edu

**Keywords:** luminescence, cellular luminescence imaging, diagnose, lanthanide, two-photon absorption

## Abstract

The use of luminescence in biological systems allows one to diagnose diseases and understand cellular processes. Molecular systems, particularly lanthanide(III) complexes, have emerged as an attractive system for application in cellular luminescence imaging due to their long emission lifetimes, high brightness, possibility of controlling the spectroscopic properties at the molecular level, and tailoring of the ligand structure that adds sensing and therapeutic capabilities. This review aims to provide a background in luminescence imaging and lanthanide spectroscopy and discuss selected examples from the recent literature on lanthanide(III) luminescent complexes in cellular luminescence imaging, published in the period 2016–2020. Finally, the challenges and future directions that are pointing for the development of compounds that are capable of executing multiple functions and the use of light in regions where tissues and cells have low absorption will be discussed.

## 1. Introduction

The use of luminescence in biological systems allows one to diagnose and understand cellular processes [1,2,3,4,5,6,7,8]. Luminescent labels, such as organic dyes [4,9,10], transition metal complexes [8,11,12,13] and nanoparticles [1,2,3], are known, yet photobleaching and aggregation in the case of the organic dyes, as well as short emission lifetimes, and narrow Stokes shifts, limit their application.

**Figure 1:**
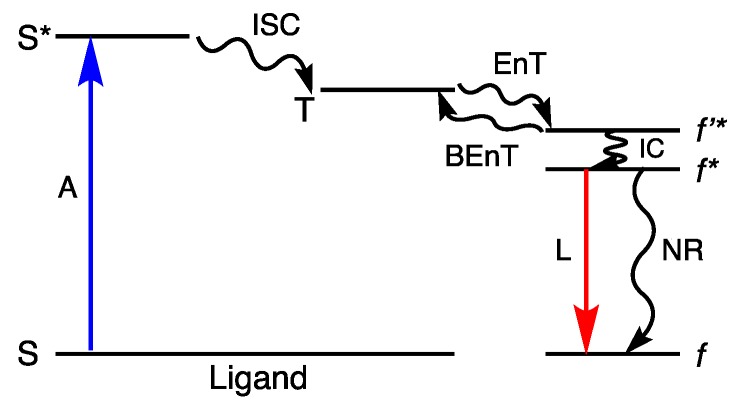
Energy level diagram illustrating the antenna effect. A is absorption, ISC intersystem crossing, EnT energy transfer, BEnT back-energy transfer, L luminescence, NR non-radiative pathways, S designates levels with singlet multiplicity and T levels with triplet multiplicity.

Lanthanide(III) (Ln^III^) ions are very attractive for application in cellular luminescence imaging [14,15,16,17,18,19,20,21,22,23,24,25,26] due to long emission lifetimes, which enable time-gated detection and thus increased signal-to-noise ratio, and narrow emission bands. As the emission is due to parity-forbidden *f*–*f* transitions, a chromophore bound to the metal ion is used as sensitizer; it absorbs energy and transfers it to the Ln^III^ ion, which then emits light (Figure 1) [24,27,28]. Soini and Hemmilä were the first ones to report on the use of the long-lived Ln^III^ emission in bioimaging [29]. That was followed by decades of contribution from Bünzli′s research group to the establishment and applicability of Ln^III^ compounds in bioimaging [20,21,30]. Since then, the use of Ln^III^ complexes in bioimaging has flourished, and several examples are found in the literature [16,17,31,32,33].

For use in cellular luminescence imaging, the Ln^III^ complexes have to meet the following requirements: water-solubility, thermodynamic stability, absorption band at or above 405 nm, high brightness, and excited state lifetime in the micro- or milliseconds range [26]. Eu^III^ is still the most used Ln^III^ in bioimaging due to its luminescence lifetime in the millisecond range, and bright emission in the red region of the electromagnetic spectrum, that allows time-gated detection in a region in which the cells and tissues have low scattering [34].

Most recent developments in Ln^III^ luminescent complexes in the broad field of luminescence imaging are focused on systems capable of luminescence and sense a biological relevant species [35,36,37], development of compounds that are capable of imaging and therapy [31,38,39], and the use of excitation and/or emission in a region where there is low scattering by cells and tissues [15,32,40]. All those developments are geared towards compounds that are capable of executing multiple functions, which means a decrease in the cost, more results obtained with a single compound, and the use of less energetic radiation to avoid cell or tissue damage.

This review aims to provide a background in luminescence imaging and lanthanide spectroscopy and discuss selected examples of recent literature on lanthanide(III) luminescent complexes in cellular luminescence imaging, published in the period 2016–2020. For detailed information about lanthanide luminescence, internalization processes of Ln^III^ complexes, Ln^III^ bioconjugates, Ln^III^ NIR luminescence imaging, molecular upconversion systems, and upconversion nanoparticles the reader is referred to other reviews [24,28,41,42,43,44,45,46,47,48,49]. Finally, the challenges and future directions that are pointing for the development of compounds that are capable of executing multiple functions, and the use of light in regions where tissues and cells have low absorption will be discussed.

## 2. Luminescence Imaging

The basic principle of luminescence imaging is to excite a volume of a sample containing a photoluminescent label and collect the light emitted. The excitation of the sample is achieved with light coming from the top, in a setup called inverted microscope, or from the bottom, in a setup called upright microscope. Inverted microscopes are recommended for samples fixed in a glass slide, and upright microscopes for live samples due to closer proximity between lenses and sample in the former. The two used methods to image biological samples using photoluminescent labels are widefield (WF), and confocal (CF) microscopy [50].

**Figure 2:**
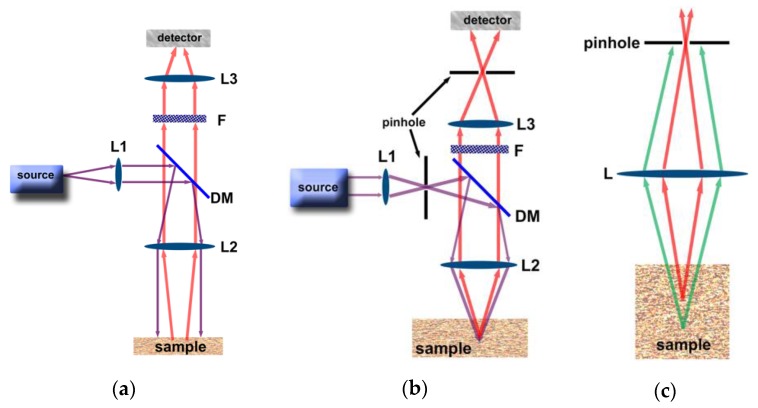
(**a**) WF and (**b**) CF microscope setup. (**c**) Exclusion of out-of-focus light by the pinhole in a CF setup. L indicates lens, DM dichroic mirror, F filter, the purple and red lines indicate excitation and emission, respectively, and the green line indicates emission coming from out-of-focus.

In WF microscopes, the excitation light is first collimated by a set of lenses (L1), reflected by a dichroic mirror (DM), and excite the sample (Figure 2a). The resulting emission passes through the dichroic mirror (DM), filter (F), and a lens (L3) focuses it on the detector that transforms the photons in the electrical signal, Figure 2a. The advantage of the WF microscopy is that it allows the use of versatile excitation sources such as Hg- (230–600 nm), Xe-arc lamp (250–1000 nm), or LEDs that cover a wide range of wavelengths. The downside of this system is that a large area of the sample is excited, resulting in undesirable background emission that causes a decrease in the signal-to-noise ratio. Also, Hg- and Xe-arc lamps have a low lifetime, ~200–500, and ~400–100 h, respectively, and an excessive amount of heat is generated, which requires special housing and ventilation. That is one of the reasons that LEDs have become popular. They have higher lifetimes (~10,000–100,000 h), generate a negligible amount of heat, and have output light intensity comparable to Hg- or Xe-arc lamps [50,51].

In CF microscopes, the excitation light is first collimated by a set of lenses (L1), passes through a pinhole, it is then reflected by a dichroic mirror (DM), focalized by lenses (L2), and excite a specific volume of the sample (Figure 2b). The resulting emission passes through the dichroic mirror (DM), filter (F), it is then focused by a lens (L3) to the pinhole and gets to the detector, Figure 2b [52,53,54]. In this setup, all the excitation light is focused on a small volume of sample, which increases the light intensity, and only light emitted from that specific point is allowed to get to the detector. The pinhole is essential in the CF system, as it excludes any emission that is not from the focal point, Figure 2c. CF setups allow a better resolution with increased signal-to-noise ratio and construction of 3D images. The downside of this system is the cost, and the possibility to use only lasers as the excitation source, which constrains the possible wavelengths (405, 440, 488, 514, 568, 635 and 685 nm, are the most common). In both WF and CF microscope setups described above, the same lens is used to both excite and collect the emission from the sample. Thus, the two systems receive the epi-fluorescence name.

**Figure 3:**
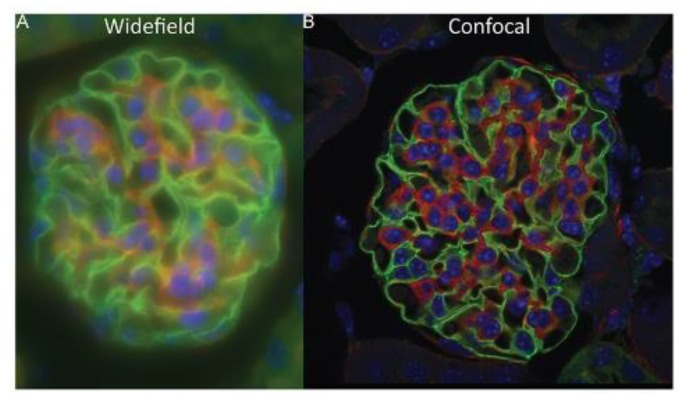
Comparison between luminescence images obtained using a WF (**A**) and a CF (**B**) microscope setups. Reproduced with permission from Elsevier [55].

The comparison between luminescence images obtained by WF and CF microscope setups is shown in Figure 3. The advantages and disadvantages of each setup are summarized in Table 1.

## 3. 4*f*-4*f* Electronic Transitions

**Figure 4:**
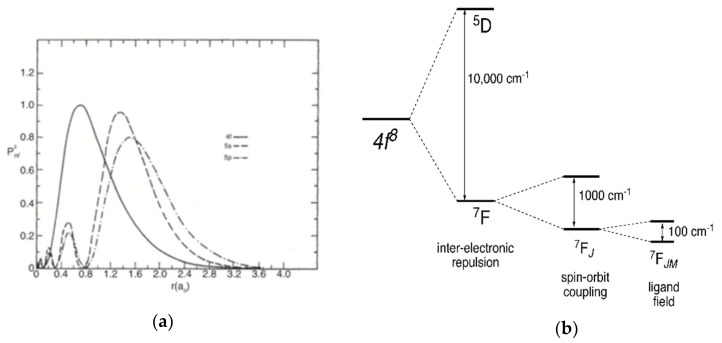
(**a**) Radial probability distribution of 4f, 5s and 5p electrons for Pr^III^ Reproduced with permission from Elsevier [58]); (**b**) Magnitude of the inter-electronic repulsion, spin-orbit coupling and ligand field of the 4*f^6^* configuration of a Ln^III^ ion.

Luminescence is the spontaneous emission of radiation from electronically or vibrationally excited species not in thermal equilibrium with their environment [56]. The characteristic 4*f*-4*f* electronic transitions of Ln^III^ are sharp due to the small Stokes shift caused by the core nature of the 4*f* electrons, shielded from the coordination environment by the 5*s* and 5*p* electrons, which minimizes the perturbation of the 4*f* electrons by the ligand field (Figure 4a,b) [57]. As a result of the shielding and high atomic number, the inter-electronic repulsion has a magnitude of ~10^4^ cm^−1^, while the spin-orbit coupling and ligand field have magnitudes of ~10^3^ and ~10^2^ cm^−1^, respectively. Thus, the splitting of the energy levels of the Ln^III^ ions is dominated by the first one, as shown in Figure 4b [57].

**Figure 5:**
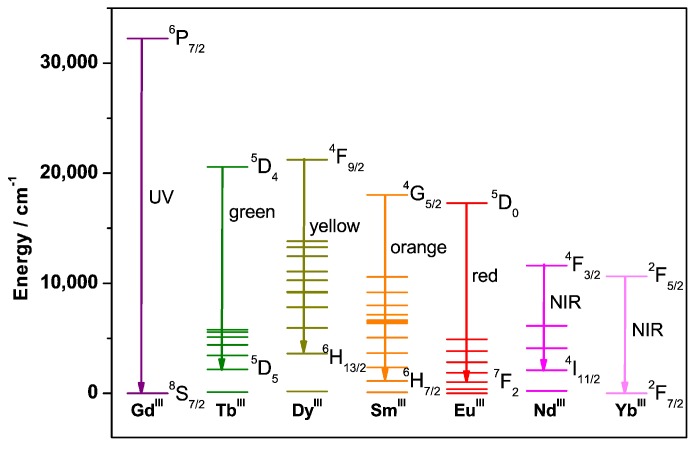
Main electronic transitions of the Gd^III^, Tb^III^, Dy^III^, Sm^III^, Eu^III^, Nd^III^, and Yb^III^ ions.

The energies of the transitions are therefore characteristic of each Ln^III^ ion, and the most intense transitions are located, for example, in the UV (Gd^III^), visible (Tb^III^—green, Dy^III^—yellow, Sm^III^—orange, Eu^III^—red) or near infra-red (Nd^III^ or Yb^III^), as illustrated in Figure 5.

The unique nature of the 4*f*-4*f* electronic transitions is examined in the seminal publication by Van Vleck [59], in which he discusses the possible mechanisms for the 4*f*-4*f* electronic transitions, namely magnetic dipole ($\vec{M}$), electric quadrupole ($\vec{Q}$) and electric dipole ($\vec{P}$), as summarized in Table 2.

The magnetic dipole operator depends on the coupling between the magnetic orbital and spin moments and explains part but not all the observed transitions. The 4*f*-4*f* transitions are allowed by electric quadrupole, however, the calculated oscillator strengths (10^−11^) are lower than the experimental ones (10^−7^). The electric dipole mechanism cannot connect states with the same parity (for example, *f*-*f*) in the presence of an inversion center, also known as the Laporte or parity rule. However, in an asymmetric ligand field, the inversion center is absent, and the Laporte rule is relaxed. This mechanism is known as forced electric dipole and can be used to explain the 4*f*-4*f* electronic transitions and the observed experimental oscillator strengths [60,61,62]. The Laporte rule can be demonstrated using group theory. For this example, the symmetry point group *O* will be considered to predict if a given 4*f*-4*f* transition is allowed or not. According to the Wigner-Eckart theorem (Equation (1)), if the direct product between the initial state (Γ*_i_*) and electric dipole operator (Γ*_µ_*) irreducible representations contains the final state ir.educible representation Γ*_f_*, then the transition is allowed [63]:

Γ*_i_* × Γ*_µ_* ⊂ Γ*_f_*, (1)

where Γ is the irreducible representation associated with the initial (*i*) and final (*f*) states, and the electric dipole operator (*µ*).

**Figure 6:**
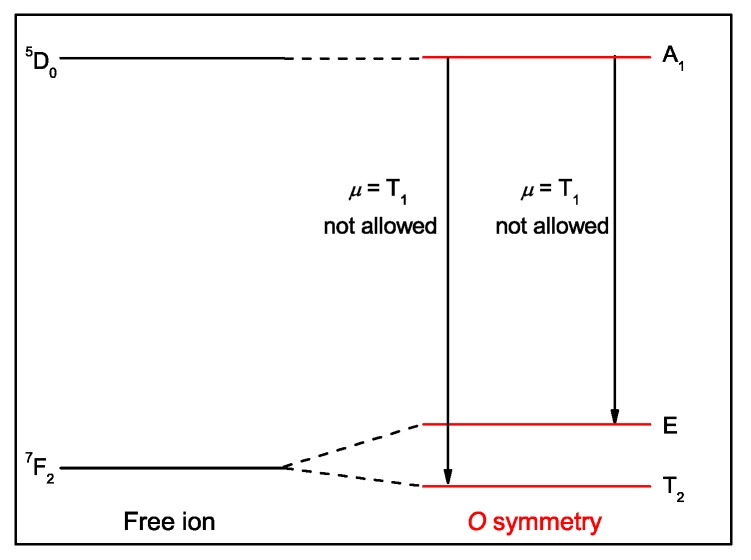
Energy level scheme showing the electronic levels ^5^D_0_ and ^7^F_2_ in the free ion (black), and the symmetry point group *O* (red), and transition probability for the ^5^D_0_ → ^7^F_2_ electronic transition.

In this case, the direct product between the ^5^D_0_ and electric dipole irreducible representations is, *Γ*_5D0_ × Γ*_µ_*= A_1_ *×* T_1_ = T_1_; thus, only transitions to final states that have the A_1_ representation will be allowed. Therefore, in the symmetry point group *O*, the ^5^D_0_ → ^7^F_2_ transition is not allowed, Figure 6. For more details regarding the symmetry rules and the number of allowed transitions, the reader is referred to the literature [63,64,65].

The Laporte forbidden nature of the *f*-*f* transitions results in a low absorption coefficient, ~1–10 mol^−1^ L cm^−1^, and long excited state lifetimes, in the range of micro to milliseconds. The limitation imposed by the low molar absorptivity is circumvented by coordinating a chromophore to the Ln^III^. The chromophore functions as the sensitizer, and this process is known as the antenna effect. In a Ln^III^ coordination complex, the light is absorbed by an organic chromophore (through S → S* transitions); after inter-system crossing (ISC) the energy is transferred from the triplet level T of the ligand to the Ln^III^ excited level f*, and finally emitted through the characteristic 4*f*-4*f* transitions, as shown in Figure 1. The influence of the ligand′s triplet level T energy, of the symmetry of the complex and the donor-acceptor distance on the luminescence efficiency, has been extensively described [28,66,67]. Charge transfer states such as ligand-to-metal (LMCT) and intra-ligand charge (ILCT) can also contribute to the energy transfer process [28,68,69].

### 3.1. Quantum Yield of Sensitized Emission ($\Phi_{L}^{Ln}$) and Brightness (B_λ_)

The characterization of Ln^III^ ion complexes for emission applications involves quantification of the emission efficiency of the compound, also called the quantum yield of sensitized emission, $\Phi_{L}^{Ln}$. In the case of Eu^III^, the intrinsic quantum yield, $\Phi_{Ln}^{Ln}$, is easily accessible experimentally (*vide infra*) and thus also often reported. $\Phi_{Ln}^{Ln}$ measures the ability of a given ligand system to protect the emissive levels from non-radiative deactivation.

The intrinsic quantum yield is equivalent to the emission efficiency using direct *f*-*f* excitation. Technically, it is possible to measure the $\Phi_{Ln}^{Ln}$ using an integrating sphere for samples in the solid-state if the ligand band does not overlap with the *f*-*f* transitions [68,70,71]. However, depending on the non-radiative and radiative rates, the measurement using an integrating sphere results in errors up to 60% [68,71]. Therefore, the determination of $\Phi_{Ln}^{Ln}$ using Equation (2) leads to the most trustable results:

(2) $$\Phi_{Ln}^{Ln}=\frac{A_{rad}}{A_{tot}}$$

where *A_tot_* is the total radiative emission rate (*A_tot_ = 1*/*τ_obs_* *τ_obs_* is the experimentally determined emission lifetime) and *A_rad_* is the radiative emission rate, determined using Equation (3) [72]:

(3) $$A_{rad}=\overset{i}{\underset{i=1}{\sum }}A_{i}=\frac{64\cdot \pi^{3}\cdot \nu_{i}{}^{3}}{3\cdot \left(2\cdot J+1\right)\cdot h\cdot c^{3}}\cdot {\left[\chi_{ed}\cdot S_{ed}+\chi_{md}\cdot S_{md}\right]}_{i}$$

*ν_i_* is the frequency of the transition, *J* is the quantum number of the initial state, *χ_ed_* and *χ_md_* are the Lorentz local field corrections, *S_ed_* and *S_md_* are the strengths of the transitions. *ed* and *md* stand for electric dipole and magnetic dipole, respectively. For the particular case of Eu^III^ the calculation of *A_rad_* can be done using the emission spectra, and Equation (3) simplifies to Equation (4) [65]:

(4) $$A_{rad}=A_{MD,\;0}\times n^{3}\times \frac{I_{tot}}{I_{MD}}$$

where *A_MD, 0_* is the coefficient of spontaneous emission for the ^5^D_0_ → ^7^F_1_ magnetic dipole transition (14.65 s^−1^), *n* is the refractive index of the solution, *I_tot_* and *I_MD_* are the integrated area of the whole emission spectra and of the ^5^D_0_ → ^7^F_1_ transitions, respectively.

In the case of the other lanthanides(III), there is no “pure” magnetic dipole transition which means that the absorption spectra must be used to calculate *A_rad_*. More details about the equations and the method use to obtain *A_rad_* is described by Sigoli and co-workers [73].

The experimental determination of the quantum yield of sensitized emission can be done through absolute or comparative methods. Measurement using the absolute method involves the use of an integrating sphere, to determine the ratio of photons emitted by the sample inside the integrating sphere to incident photons, as outlined in Equation (5) [27]:

(5) $$\Phi =\frac{\left(I_{sample}-I_{empty}\right)}{\left(L_{empty}-L_{sample}\right)}$$

where *I* is the intensity of emitted light and *L* is the scattering of incident radiation observed. *sample* and *empty* stand for integrating sphere with and without the sample, respectively.

The comparative method involves the determination of the quantum yield using a standard. A list of different standards is described in the literature [74]. When using a standard, it is desirable to excite sample and standard at the same wavelength, and it is desirable that sample and standard have emission bands in the same region of the spectrum, to account for the wavelength-dependent instrument response. To overcome potential problems with sample concentrations outside the Lambert-Beer regime, the dilution method is often used. Several solutions with different concentrations of sample and standard are prepared and the overall quantum yield is then determined with Equation (6):

(6) $$\Phi_{x}=\frac{n_{x}^{2}\cdot I_{std}\cdot Grad_{x}}{n_{std}^{2}\cdot I_{x}\cdot Grad_{std}}\cdot \Phi_{std}$$

where *n* is the refractive index, *I* is the intensity of the excitation source, *Grad* are the slopes of the plots of integrated emission spectra against absorbance of each solution for sample and standard, and *Φ_std_* is the quantum yield of the standard. A list with several standards and its excitation and emission wavelengths can be found in the literature [74].

In luminescence imaging, brightness (*B*_λ_) is an essential parameter to be considered. High brightness is desirable to obtain images with low background noise in short periods of time. The emission brightness (*B*_λ_) is determined using Equation (7):

(7) $$B_{\lambda }=\varepsilon_{\lambda }\times \Phi_{L}^{Ln}$$

where *ε* is the molar absorptivity (or molar extinction coefficient), and $\Phi_{L}^{Ln}$ is the quantum yield, both determined at the wavelength *λ*. To maximize the brightness, a combination of high molar absorptivity coefficients and high quantum yield is necessary.

### 3.2. Deactivation of the Ln^III^ Excited State

**Figure 7:**
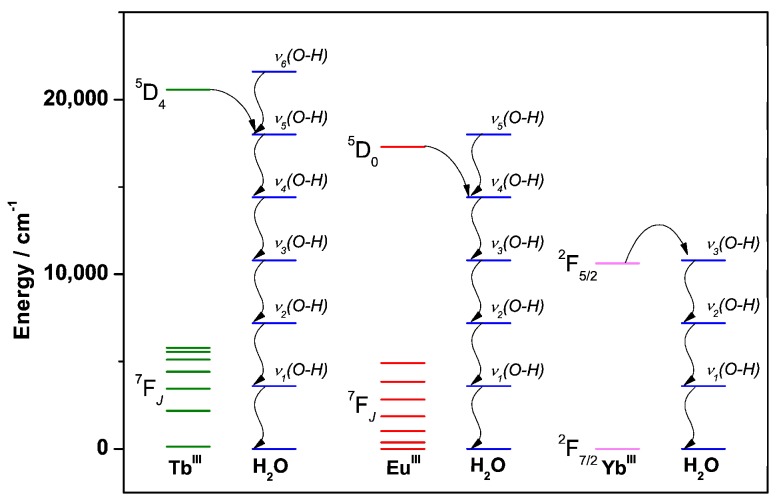
Energy diagram showing the electronic levels of Tb^III^ (green), Eu^III^ (red), and Yb^III^ (light pink), and the phonons for the water molecule O-H vibrations (blue).

The Ln^III^ emission intensity is sensitive to non-radiative deactivation processes such as back-energy transfer, thermal deactivation, and deactivation by vibrational coupling between the Ln^III^ excited level and coordinated solvent molecules. Figure 7 shows the electronic levels for Tb^III^, Eu^III^ and Yb^III^, and the phonons for the water molecule O–H vibrations *ν*(O–H) ~3600 cm^−1^). As shown in Figure 6, quenching of the ^5^D_4_ Tb^III^ and ^5^D_0_ Eu^III^ excited levels require vibrational coupling with 5–6, and 4–5 phonons, respectively, while the ^2^F_5/2_ Yb^III^ excited level only requires vibrational coupling with three phonons. The ease in quenching the Yb^III^ excited level is one of the challenges to overcome in developing Yb^III^ complexes for luminescence imaging.

The number of coordinated water molecules (*q*) to Eu^III^, Tb^III^, and Yb^III^ ions is correlated with the emission lifetime (*τ*) and can be determined using the Equations (8)–(10) [75,76], respectively:

(8) $$q_{Eu}=1.1\times \left(\frac{1}{\tau_{H2O}}-\frac{1}{\tau_{D2O}}-0.31\right)$$

(9) $$q_{Tb}=4.2\times \left(\frac{1}{\tau_{H2O}}-\frac{1}{\tau_{D2O}}\right)$$

(10) $$q_{Yb}=1\times \left(\frac{1}{\tau_{H2O}}-\frac{1}{\tau_{D2O}}-0.20\right)$$

where *τ* is the emission lifetime measured in H_2_O and D_2_O.

**Figure 8:**
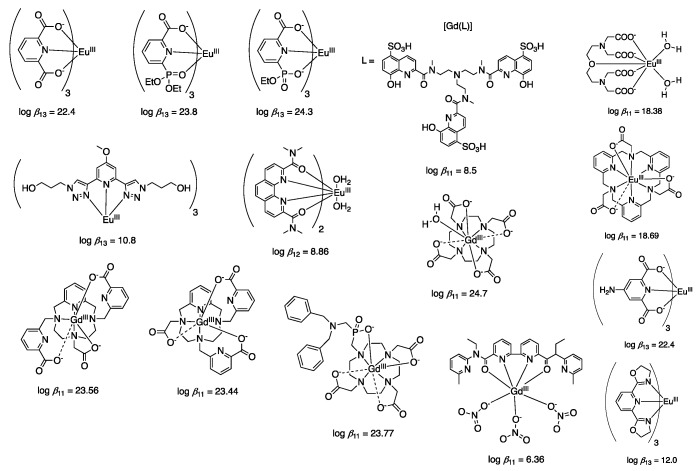
Structure of several Ln^III^ complexes along with their stability constants (*β*) [31,78,79,80,81,82,83,84,85,86,87,88].

The stability constant *β*, or more commonly its log, log(*β*), is a number that correlates with the stability of the Ln^III^ complex in solution. A high value of log(*β*) means that the concentration of free Ln^III^ and free ligand in solution is small; in other words, it means stability in solution. A wide variety of Eu^III^ and Gd^III^ complexes, along with their log(*β*) values are shown in Figure 8 [27,28,77]. Eu^III^ and Gd^III^ were chosen mainly because of the number of reports available, and because those elements are in the middle of the Ln^III^ series; thus, they are a good representation of the whole series. Usually, tri- or multi-dentate negatively charged ligands form Ln^III^ complexes with high stability constants due to the thermodynamic stability yielded by the chelate bonding, and strong ionic interaction between charged ligand and Ln^III^, respectively. As shown in Figure 8, uncharged ligands tend to form less stable Ln^III^ complexes compared with the charged ones. General guidelines can be drawn from the compilation of different stability constants for different Ln^III^ complexes. However, the presence of competing cations, the size of the ligands, and the coordination strength of the solvents are factors that must be taken into account as well. For biological applications, Ln^III^ complexes containing multi-dentate ligands and coordination numbers of nine or higher are preferred due to the high stability in aqueous solution.

### 3.3. Cell Lines Abbreviations and Ligand Structures

**Figure 9:**
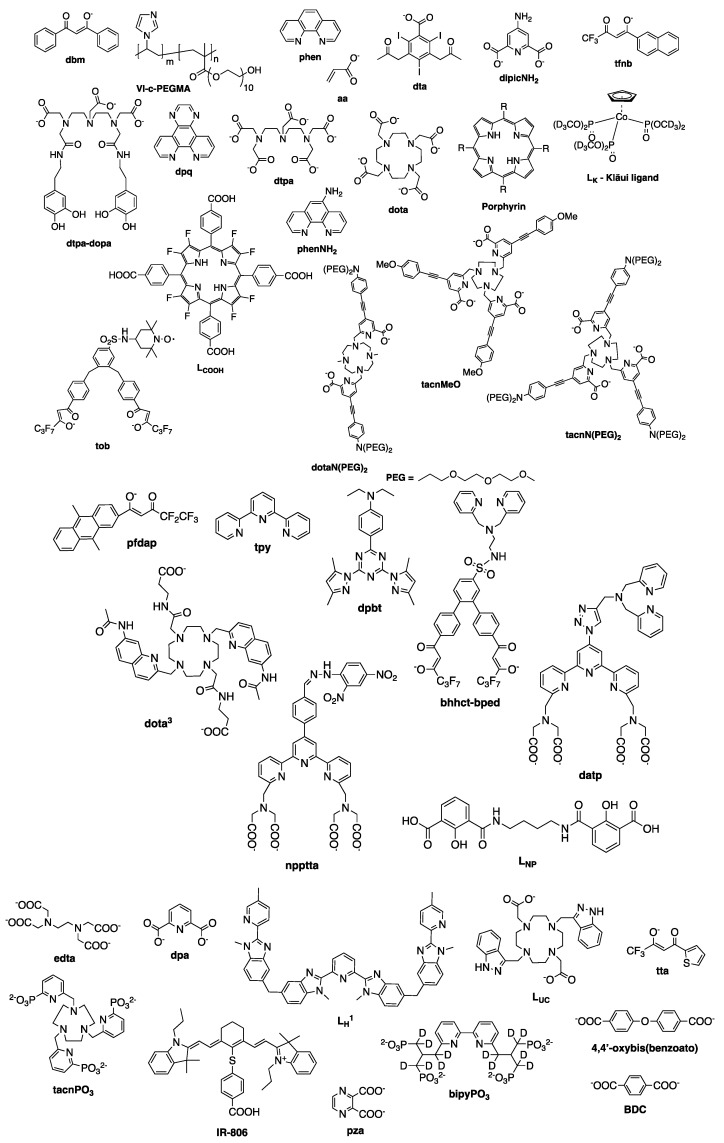
Structures of the ligands mentioned throughout this review.

The cell line abbreviations and ligand structures mentioned along this review are shown below on Table 3, and Figure 9.

## 4. Ln^III^ Complexes in Bioimaging

### 4.1. Nanoparticles and Polymers Systems Functionalized with Ln^III^ Complexes in Bioimaging

Nanoparticles (NPs) are a versatile platform due to their facile synthesis and easy functionalization of the surface to achieve multiple functionalities, for example, luminescence imaging and therapy [89,90]. The low cell penetrability, low molar extinction coefficient, and absorption band in the deep UV region of the electromagnetic spectrum are however limiting factors for the use of NPs in luminescence imaging [91]. Those are circumvented by functionalizing its surface with cell receptors that facilitate cell recognition and uptake, and Ln^III^ complexes, that improve the absorption and emission of light [92,93,94,95,96,97], respectively. For example, surface functionalization of hydroxyapatite NPs (HNPs) with [Eu(dbm)_3_(H_2_O)_2_] complexes yielded a system with low cytotoxicity and capable of luminescence imaging HeLa cells [92,93,94]. Although the system mentioned above is biocompatible, the cell uptake is low, resulting in the use of higher concentrations to obtain the luminescence images. Surface functionalization with folic acid (FA), a receptor overexpressed in cancer cells, improved the cellular uptake of the Eu^III^-HNPs by HeLa cells [95]. The use of nucleic acid-base aptamers is another strategy for improving the NPs cell uptake due to its low cost, strong interaction, and specificity towards cancer cells [98]. Bioconjugation of Ln^III^ complexes, protected by a silica shell, with the aptamer Sgc8 using glutaraldehyde or succinic anhydride and EDAC/Sulfo-NHS resulted in a system that has a strong affinity for CCRF-CEM and Jurkat cells [96,97].

**Figure 10:**
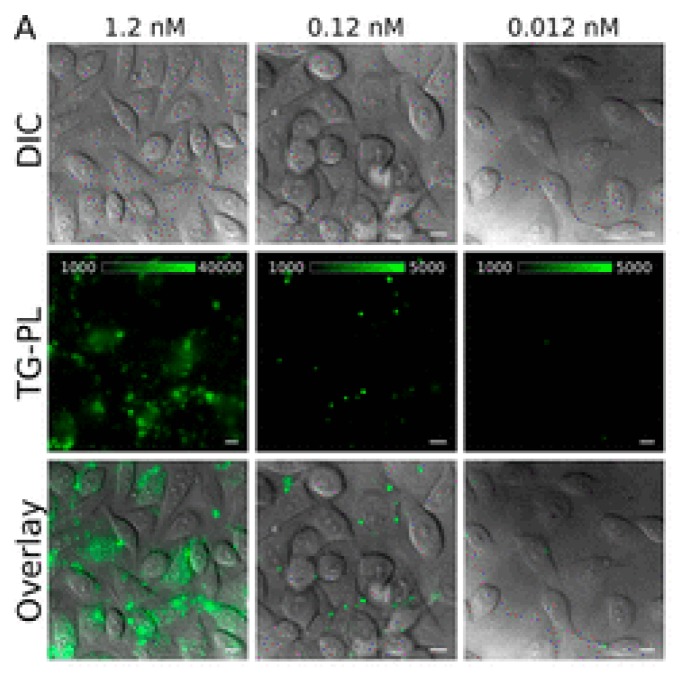
Time-gated luminescence imaging of HeLa cells incubated with the NP-L_NP_ hybrid system for 24 h. Concentration = 1.2 (left column), 0.12 (middle column), and 0.012 nM (right column). The first, second, and third rows correspond to the bright field, time-gated luminescence, and overlay images. Reprinted with permission from [100]. Copyright (2020) American Chemical Society.

Coating Ln^III^ complexes with silica shells [96,97], use of heterobimetallic Ir^I^/Eu^III^ [99], and decoration of NPs surface with chromophores [100] are strategies used to improve the emission intensity of NPs. For example, Ir/Eu heterobimetallic complexes were trapped inside mesoporous silica nanoparticles (MSN) to improve water solubility. The system showed emission quantum yield of 55.2%, low cytotoxicity in the concentration range 0–200 mg mL^−1^ and was used for luminescence imaging of HeLa cells [99]. Decoration of Ln^III^ NPs with chromophores is another strategy for improving the Ln^III^ emission intensity, and for protecting the Ln^III^ against solvent molecules coordination [100]. Using this strategy, La_0.9_Tb_0.1_F_3_ NPs decorated with 3,3′-((butane-1,4-diylbis(azanediyl))bis(carbonyl))bis(2-hydroxybenzoic acid)–L_NP_–were used in luminescence imaging of HeLa cells (Figure 10) [100].

Polymeric systems have high absorption cross-section, high photostability, and, similar to NPs, offers the possibility of multi functionalities through the attachment of different compounds throughout the polymeric chain [101,102,103,104,105]. The broad emission bands and the low emission lifetimes are disadvantages of polymeric systems for use in luminescence imaging. Coordination of Ln^III^ in the polymer structure results in systems with narrow emission bands, and emission lifetime in the microsecond to millisecond range that is suitable for time-gated imaging [106,107,108,109,110,111]. Biocompatible polymers such as polysiloxanes, imidazole-based polymers, and polymeric sugar chains functionalized with Ln^III^ are widely used in luminescence imaging [107,108,109]. Imidazole-based polymers are particularly advantageous due to the capability of bonding to Ln^III^ using the nitrogen atom from the imidazole moiety. Thus, in this kind of system extra steps to add Ln^III^ coordinating capabilities are not required. For example, self-assembled imidazole polymers (VI-*c*-PEGMA) coordinated to Dy^III^ and coated with silica was found to be chemically stable and successfully used in the luminescence imaging of L929 cells [108]. Although not specified, it is assumed that the images were obtained using excitation centered at the Dy 4*f*-4*f* transitions. Sugar polymeric chains are an alternative in the search for more biocompatible polymers due to their ease diffusion in cells and in the body [112]. Natural sugar polymeric chains modified with the [Tb(dota)] complex, were successfully used in time-gated images of arteries yielding results comparable with the magnetic resonance imaging (MRI) using similar Gd^III^ complexes [109].

Systems capable of executing multiple functions, also called multimodal systems, are desirable due to the possibility of obtaining more information using a single system [113,114,115]. For example, NPs functionalized with the [Eu(aa)_2_(dta)(phen)] complex can be used not only in luminescence imaging but also in X-ray computed tomography imaging (CT) due to the high X-ray absorption cross-section of Eu^III^ [116]. As discussed above, NPs and polymers functionalized with Ln^III^ complexes are dynamic systems for application in luminescence imaging. However, the internalization of NPs in cells is difficult due to the size in the range from tenths to thousands of nanometers, resulting in undesired accumulation in the body, and limiting in vivo applications [91]. The use of molecular systems, namely Ln^III^ complexes, provides the control of the spectroscopic properties at the molecular level, and tailoring of the ligand structure that adds sensing and therapy capabilities, for example.

### 4.2. Visible Emitting Ln^III^ Complexes in Bioimaging

Control of the spectroscopic and chemical properties at the molecular level and the higher cell penetrability, due to the small size, of Ln^III^ luminescent complexes, are advantages for use in luminescence imaging of biological systems. The formation of the Ln^III^ complexes inside the cells is the most straightforward strategy used in luminescence imaging [117,118]. For example, treatment of Hepg2 cells with Eu(NO_3_)_3_ produced a luminescent Eu^III^ complex that is not observed using the healthy L02 cell lines [117]. Although there is selectivity towards cancer cells, the identity of the ligands bonded to Eu^III^ could not be figured out, and only a possible mechanism of formation involving NADPH was proposed. Attempts to get more information about the identity of the complexes formed in CHO cells treated with Eu^III^ or Tb^III^ acetate were made by Sørensen and co-workers, using a state-of-the-art confocal microscope [118]. The comparable intensities of the ^5^D_0_ → ^7^F_1_ and ^5^D_0_ → ^7^F_2_ transitions in the emission spectra suggested that the Eu^III^ is in a high symmetry coordination environment [118]. The luminescence images also showed Ln^III^ accumulation in the glycocalyx that points to bonding with specific components of it such as sugars [118].

**Figure 11:**
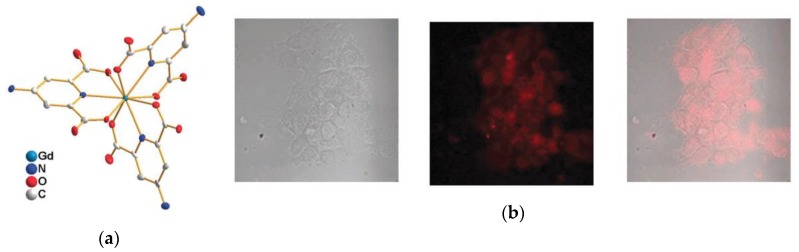
(**a**) Single crystal X-ray structure of the [Gd(dipicNH_2_)_3_]^3−^ complex and (**b**) Bright field, luminescence, and overlay imaging of the NG97 cells after 12 h of incubation with [Eu(dipicNH_2_)_3_]^3−^. Reproduced from [31] with permission from the Royal Society of Chemistry.

Spontaneous internalization of Ln^III^ complexes in cells is unpredictable, and effort has been made to determine the correlation structure-cell uptake [21,42,119,120,121,122,123,124,125,126,127]. The usual mechanism of cellular uptake of low molecular weight complexes is endocytosis [42]. In this mechanism, the complex interacts with the membrane forming vesicles that are responsible for the internalization of the Ln^III^ complexes in the cell [42]. Thus, shape [119,120,121,122,123,124,125], chirality [128], and charge [21,42,127] are some of the factors that influence cell uptake. A thorough study conducted by Parker′s research group, using dota- and triazacyclonane-derivatized Ln^III^ complexes, concluded that the mechanism of cell uptake involves recognition of the Ln^III^ complex by proteins. Thus, the shape and the chirality of the complex are factors that determine cell uptake [119,120,121,122,123,124,125,128]. The charge also plays an essential factor in the cell uptake of Ln^III^ complexes. Due to the strong negative charge of the cellular membrane, Ln^III^ complexes with a positive overall charge are more likely to be internalized [21,42,127]. Although general guidelines for improving cell uptake are found, this process is sometimes unpredictable. Other studies found that change in the hydrophobicity or charge does not influence cell uptake [126]. In other cases, simple functionalization of the dipicolinato-based ligands with amino (-NH_2_) groups, yielded Eu^III^ complexes that are selectively internalized by NG97 and PANC1 cells, capable of imaging, and show moderate cytotoxicity towards those kinds of cells (Figure 11) [31].

The bioconjugation of Ln^III^ complexes with antibodies or proteins improves the cell uptake and allows targeting a specific kind of cell. The bioconjugation is achieved by reacting the amino (-NH2) or tiol (-SH) groups from a protein with isothiocyanato, chlorosulphonyl, 2,4-dichloro-1,3,5-triazinyl, or *N*-hydroxysuccinimide groups from the Ln^III^ complex [77,111,119,129]. Although bioconjugation is an expensive method, it yields luminescent compounds that have a particular target cell [130].

**Figure 12:**
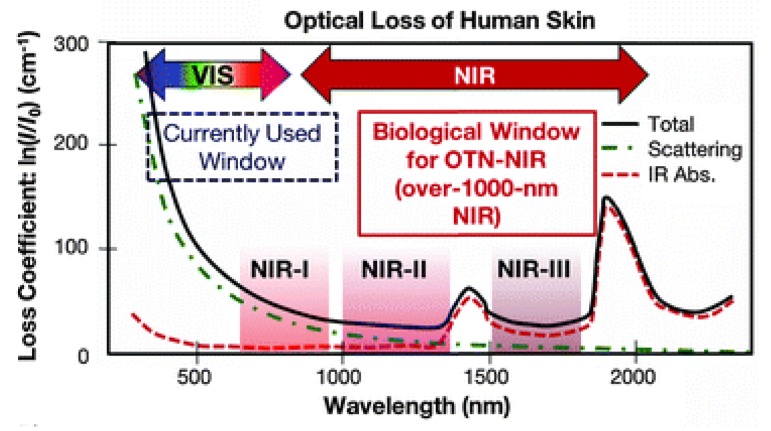
Plot “attenuation coefficient as a function of the wavelength” for human skin tissues. Reproduced from [34] with permission from The Royal Society of Chemistry.

Accumulation of Ln^III^ luminescent compounds in a specific organelle helps to unravel the different cellular processes [131,132,133]. Recent examples show that Ln^III^ complexes are found to accumulate in different regions of the cell, such as lysosome [134], nucleoli [135], cytosol [136], and primary cilium [137]. The ligand structure plays an essential role in directing the specific organelle that the Ln^III^ complexes accumulate. For example, functionalization of the ligands with triphenylphosphonium, morpholine, or methyl phenyl sulfonamide yielded accumulation of Ln^III^ luminescent complexes in the mitochondria, lysosomes, and endoplasmic reticulum, respectively [138,139,140]. Dopamine-functionalized complexes [Ln(dtpa-dopa)(H_2_O)] (Ln = Eu^III^ or Tb^III^), were found to accumulate in the cytosol of HeLa and Neuro-2 cells, with low cytotoxicity [136] while the [Ln(tfnb)_3_(dpq)] (Ln = Eu^III^ or Tb^III^) complexes containing planar aromatic ligands were found to accumulate in the nucleoli [135]. In this case, the strong interaction between [Ln(tfnb)_3_(dpq)] (Ln = Eu^III^ or Tb^III^) complexes and DNA, and extensive photoinduced DNA damage (*λ_exc_* = 364 nm) were used to kill H460 cells [135]. The examples mentioned above highlight the potential of molecular Ln^III^ systems in luminescence imaging. Although successful, emission in the visible is scattered by cells and tissues; thus, luminescent Ln^III^ complexes with emission in the biological window—a region where cells and tissues have low absorption—is needed (Figure 12).

### 4.3. NIR Emitting Ln^III^ Complexes in Bioimaging

NIR emitting Ln^III^ complexes are being recently applied in luminescence imaging due to its emission in the biological window. There are two significant challenges for developing NIR emitters for use in luminescence imaging, the first one is the low instrumental sensitivity in the NIR, and the second one is to overcome the high non-radiative rates caused by vibrational coupling with O-H vibrations, mentioned in Section 3.2, reflecting in low emission quantum yields, and low brightness compared with visible emitting Ln^III^ [40,141].

**Figure 13:**
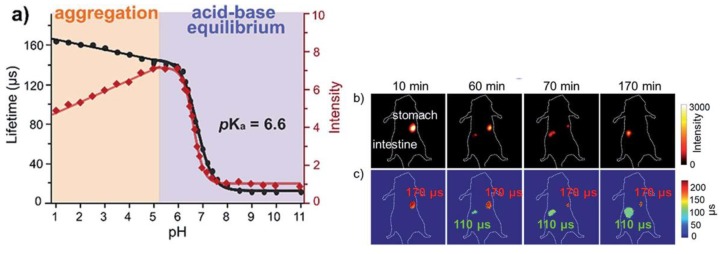
(**a**) Plot Yb^III^ emission lifetime (black trace) and Yb^III^ emission intensity (red trace) as a function of the pH in the range 1–11. (**b**) NIR luminescence imaging and (**c**) emission lifetime imaging showing migration of the Yb^III^ complex from the stomach (pH 1–3) to the intestine (pH 6–7) [17]. Reproduced from [17] published by The Royal Society of Chemistry.

[Nd(dtpa)] and [Nd(dota)] complexes were successfully used in in vivo NIR luminescence imaging [142,143]. Although successful, due to the absence of chromophore groups in the ligands′ structure, the excitation was centered at the 4*f*-4*f* transitions that required high excitation power and longer acquisition times. To overcome this limitation, Yb^III^ complexes with porphyrin-based ligands and Kläui ligands (L_K_) have been used for in vitro and in vivo NIR luminescence imaging [16,17,25,144]. Porphyrin-based ligands have low energy singlet and triplet levels that are adequate to sensitize NIR emitting Ln^III^ [145]. The functionalization of porphyrin ligands with a benzoic acid moiety yielded Yb^III^ complexes ([Yb(L_COOH_)(L_K_)]) that are capable of sensing pH in vitro and in vivo [17]. The decrease in the Yb^III^ emission lifetime was observed in the range 5.0–9.0, and 5.0–1.0 due to photoinduced electron transfer (PET) and aggregation effects, respectively (Figure 13) [17]. The use of polymeric systems formed by 1,*n*–dihydroxyanthraquinone-functionalized polystyrene (PS) is also a strategy used for sensitizing the Yb^III^ emission and imaging HeLa cells with low cytotoxicity [146]. The use of Sm^III^ complexes is an alternative to the traditional Yb^III^ and Nd^III^ NIR emitters. Due to transitions in both the visible (^4^G_5/2_ → ^6^H_7/2_, ~600 nm) and NIR (^4^G_5/2_ → ^6^F_5/2_, ~950 nm) Sm^III^ complexes are attractive for combined Vis and NIR luminescence imaging [147].

### 4.4. Two-Photon Excitation Ln^III^ Complexes in Bioimaging

**Figure 14:**
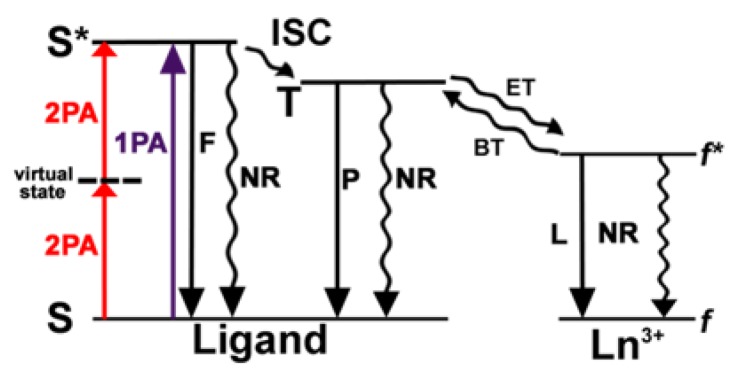
Energy level diagram illustrating the antenna effect for Ln^III^. 2PA and 1PA are the two- and one-photon absorption, F fluorescence, P phosphorescence, ISC intersystem crossing, ET energy transfer, BT back-transfer, L luminescence, NR non-radiative pathways, S states with singlet and T states with triplet multiplicity. Reprinted with permission from [40]. Copyright (2020) American Chemical Society.

Shifting the excitation towards longer wavelengths is another strategy to minimize interference from the background emission. However, the shift towards longer wavelengths results in decrease of the triplet level energy and inefficient sensitization of visible emitting Ln^III^ [148]. Two-photon absorption (2PA) [149,150,151,152,153,154] is a nonlinear process where two photons with half the energy required by the one-photon excitation (1PA) are absorbed simultaneously (Figure 14) [155,156]. As a result, the excitation wavelengths in the 2PA process are in the NIR and fall in the biological window.

Lakowicz and co-workers pioneered the sensitization of Eu^III^ emission using 2PA [157,158]. Since then, examples of the application of Eu^III^ [15,159,160,161,162], Tb^III^ [33], Dy^III^ [33], Yb^III^ [32,163], and Sm^III^ [15,163] complexes in 2P-luminescence imaging have been demonstrated. High 2P brightness (*B*^(*2*)^), one of the critical factors for obtaining good quality luminescence imaging, is achieved by the presence of charge transfer states (CT) [40,164,165], high complex rigidity [166], or use of plasmonic bands [167]. For example, 2P-sensitized emission, using *λ_exc_* = 975 nm, of the [Eu(dbm)_3_(phen-NH_2_)] complex deposited onto a glass substrate is only observed when a layer of triangular silver nano prisms is present [167].

**Figure 15:**
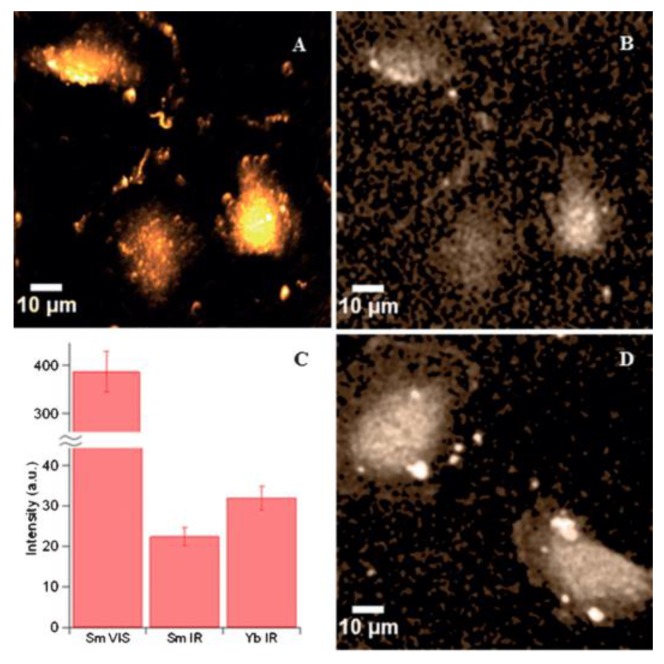
2P-luminescence imaging of T 24 cells using the [Sm(tacnMeO)]. (**A**) Visible luminescence channel. (**B**) NIR luminescence channel. (**C**) Comparison between the SmIII (visible and NIR) and YbIII (NIR) emission intensities. (**D**) 2P-luminescence imaging obtained using the [Yb(tacnMeO)] complex [163]. Reproduced from [163] with permission from John Wiley and Sons.

The use of excitation and emission in the NIR, also called NIR-to-NIR luminescence imaging, allows higher signal-to-noise ratio and luminescence imaging of deep tissues. Conventional confocal microscope setups do not allow measurement of emission in the 950–1050 nm range due to optical filtering schemes, and PMT detectors that are optimized for the visible range. By modifying the optical filtering schemes and connect an adequate NIR detector, Andraud and co-workers successfully obtained images using a combination of 2P-excitation and NIR emission (NIR-to-NIR luminescence imaging) [19]. Determination of the 3D blood capillary network in mouse brain using the NIR-emitting [Yb(tacnN(PEG)_2_)] complex validated the setup, and shortly after that, the first example of NIR-to-NIR luminescence imaging using the [Yb(dotaN(PEG)_2_)]^+^ complex was reported [32]. Due to the possibility of emitting in the visible and NIR, Sm^III^ complexes have been explored for use in luminescence imaging. For example, 2P-luminescence imaging of T24 cells in the visible and NIR was possible using the [Sm(tacnMeO)] complex (Figure 15A,B) [163]. The NIR luminescence image quality obtained was similar to the analogous Yb^III^ complex (Figure 15C,D) [163].

**Figure 16:**
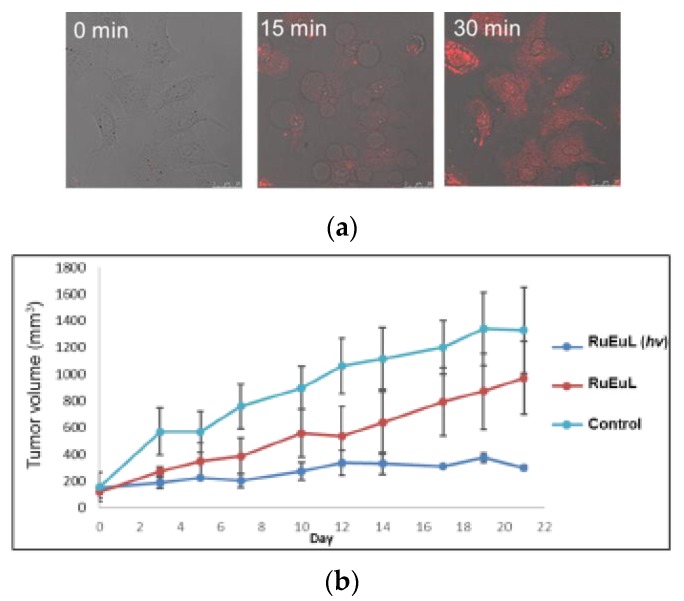
(**a**) 2P-luminescence imaging of HeLa cells at different times after irradiation at 488 nm ([complex] = 50 μM and λ_exc_ = 700 nm). (**b**) Plot tumor volume as a function of the time in the absence (aqua line), presence of the complex without light (red line), and presence of the complex with light (blue line). m_complex_ = 40 μg, λ = 488 nm. Reprinted with permission from [159]. Copyright (2017) American Chemical Society.

The addition of ^1^O_2_ generation [168,169], and DNA damage capabilities [159] to the Ln^III^ complexes opens new ways for the use of those compounds as theranostics. For example, photoactivated DNA damage and 2P-luminescence imaging capabilities are possible using the heterobimetallic [Eu(dota-py)(H_2_O)RuCl(bpy)_3_]^+^ complex [159]. Upon illumination at 488 nm, there is the release of the [RuCl(bpy)_3_]^+^ complex increasing the Eu^III^ emission intensity and also DNA damage (Figure 16) [159]. Thus, this a system that has light-activated cytotoxicity and is capable of tracking the delivery of the complex using 2P-luminescence imaging.

### 4.5. Molecular Upconversion Systems

**Figure 17:**
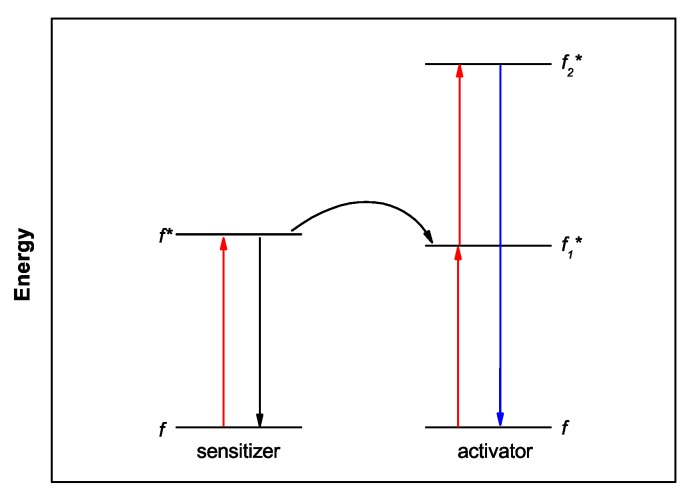
Energy level diagram illustrating the upconversion process.

Excitation of Ln^III^ complexes at longer wavelengths is achieved through non-linear optical processes, such as two-photon absorption (2PA) (*vide supra*) or cumulative effects of multiple first-order absorption phenomena, namely upconversion (UC) [170,171]. The latter can be achieved through excited-state absorption (ESA) and energy transfer upconversion (ETU), Figure 17. In the UC process, a very long-lived intermediate state is present as opposed to the 2PA one, where a short-lived intermediate state is present. This results in a higher absorption cross-section in UC, making it possible to observe this process with inexpensive and low power continuous-wave lasers [172]. In the UC process through ESA, a sensitizer ion absorbs low-energy photons, followed by energy transfer to the activator ion, which then emits in a characteristic wavelength. Yb^III^/Er^III^ [173,174,175,176], Yb^III^/Tm^III^ [113,114], and Nd^III^/Yb^III^/Er^III^ [177,178] are some of the most common sensitizer/activator systems. The challenge in developing molecular UC systems is to overcome the high non-radiative rates caused by vibrational coupling with O-H and C-H vibrations, inefficient 4*f*-4*f* excitation of the sensitizer ion, and long distances activator-sensitizer in Ln^III^ complexes that lower the energy transfer rates [43].

**Figure 18:**
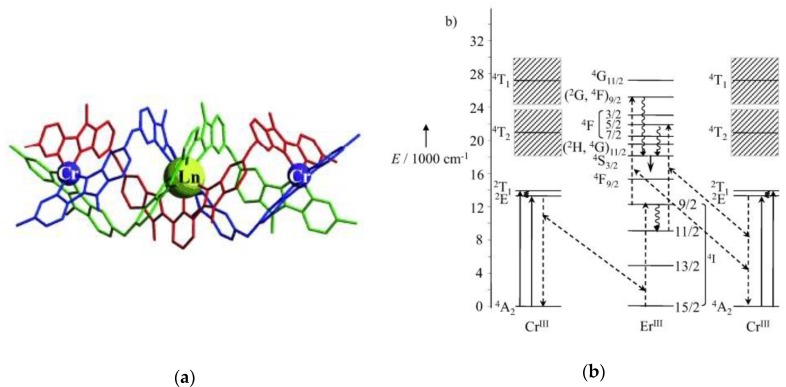
(**a**) X-ray single structure of the [CrEuCr(L_H_^1^)]_2_(CF_3_SO_3_)_18_•(C_3_H_5_N)_30_. (**b**) Energy diagram showing the energy transfer processes in the [CrErCr(L_H_^1^)]^9+^ system. Excitation solid upward arrow, internal conversion curled arrow, ETU dotted arrow. Reproduced from ref. [186] with permission from John Wiley and Sons.

The first attempt to achieve molecular UC reports back from 2005 when Faris and co-workers observed UC sensitized emission in the UV from [Nd(edta)_2_] and the blue and green from [Er(dpa)_3_]^3−^, and [Tm(dpa)_3_]^3−^. Although successful, a combination of two laser sources and high-power laser intensities (~100 kW focused on a 100 μm spot) was needed to observe the UC emission [179]. Although the intensity used was high for practical applications that encouraged researchers to design luminescent Ln^III^ complexes with improved UC properties. MOFs have a very defined solid structure with the possibility to have two or more metallic centers close to each other. Because the energy transfer process is dependent on the distance donor-acceptor, in this case, sensitizer-activator, this proximity is beneficial for improving the UC sensitization process (ETU). Jin′s research group reported a series of Ln^III^ MOFs with benzodicarboxylato (BDC) [180], pza [181], 4,4′-oxybis(benzoato) [182], in all the cases Y^III^ was used as the matrix that was doped with Yb^III^ and Er^III^ to achieve UC sensitized emission. One of the limitations of the MOFs cited above is the coordination of, at least, one solvent molecule to the Ln^III^, which increases the non-radiative rates and decreases the UC efficiency. Other Ln^III^ MOFs showing UC sensitized emission are reported [183,184,185], and the same problem mentioned above was found, the solvent coordinated to the Ln^III^ decreases the UC efficiency. Research in the development of UC sensitized emission in molecular Ln^III^ complexes using reasonable laser intensities is a field in development. Piguet and co-workers reported the first molecular system to achieve UC sensitized emission using a very elegant trinuclear Cr^III^Er^III^Cr^III^ coordinated by a helicate ligand (L_H_^1^), in frozen solution at ~30 K, Figure 18a [186]. In this system, low-intensity UC sensitized emission is achieved by Cr^III^ absorption at 750 nm (^4^A_2_ → ^2^T_1_), ETU Cr^III^ → Er^III^ followed by the characteristic 4*f*-4*f* Er^III^ centered emission in the green (^4^S_3/2_ → ^4^I_15/2_), Figure 18b [186]. In a follow-up article, the ETU mechanism was further discussed in detail, and further experimental proof of the energy pathways was provided [187]. UC sensitized emission was also demonstrated in a binuclear Cr^II^Er^III^ complex [188]. In order to avoid quenching by cross-relaxation, dilution of the [CrErCr(L_H_^1^)]^9+^ complex in a matrix of [GaYGa(L_H_^1^)]^9+^ (ratio 1:9) improved the UC emission intensity [189].

**Figure 19:**
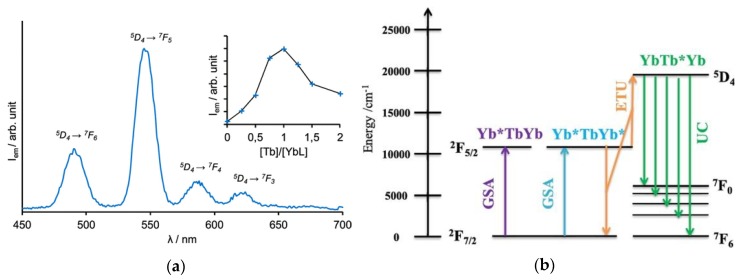
(**a**) UC emission of the [(Yb(tacnPO_3_))_2_Tb] complex. The inset shows the UC emission intensity as a function of the ratio [Tb]/[Yb(tacnPO_3_)]. [Yb^III^] = 1.25 mM, in D_2_O (pD ~7.1). *λ_exc_* = 980 nm, *p* = 1.08 W. Reprinted with permission from [194], copyright (2019) American Chemical Society. (**b**) Energy level diagram showing the UC energy transfer mechanism for the [(Yb(tacnPO_3_))_2_Tb] complex. GSA is ground state absorption, ETU is energy transfer upconversion, and UC is upconversion emission. Reproduced from [43] with permission from Elsevier.

In a quest to miniaturize the UC systems, Piguet and co-workers designed Er^III^ complexes with ligands that are capable of shielding Er^III^ from non-radiative processes due to coupling with high energy oscillators resulting in emission at room temperature in the solid-state [190]. UC sensitized emission in solution is challenging due to the efficient vibrational coupling with high energy oscillators (*vide supra*) and slow energy transfer rates between the Ln^III^ caused by dilution of the complex, which is essential in the ETU process. UC sensitized emission in D_2_O, at room temperature, was first observed using the [Er(L_uc_)]^+^ [191]. In order to decrease the distance Er^III^-Er^III^, F^-−^ ions were added to balance the charge and force the formation of a dimer. The characteristic Er^III^ UC emission in the green (^2^H_11/2_ → ^4^I_15/2_ and ^4^S_3/2_ → ^4^I_15/2_), and red (^4^F_9/2_ → ^4^I_15/2_) are observed, and the UC emission mechanism consists of GSA/ESA, where one Er^III^ center absorbs two-photons, and ETU, where there is energy transfer between two Er^III^ centers. Recently another example of UC sensitized emission in solution, at room temperature, was reported for a mononuclear Er^III^ complex [192]. Recently, UC sensitized emission of Tb^III^ was demonstrated by Charbonnière and co-workers in D_2_O solution using a system Yb^III^/Tb^III^ [193,194]. In those systems, a Yb^III^ mononuclear complex is first generated using the ligands bipyPO_3_ [193] or tacnPO_3_ [194], followed by the addition of Tb^III^ forming a supramolecular structure Yb^III^Tb^III^Yb^III^. The formation of the supramolecular structure is possible due to the coordination of Tb^III^ to the free P-O^-−^ groups. The characteristic Tb^III^ transitions (^5^D_4_ → ^7^F*_J_*; *J* = 6–0) are observed (Figure 19a) and the proposed mechanism consists of cooperative upconversion (CU) where two Yb^III^ centers populate the ^5^D_4_ excited level of Tb^III^ (Figure 19b) [194].

The low molar extinction coefficient of the 4*f*-4*f* transitions and the non-radiative rates due to the presence of C-H bonds in the structure of the ligands are factors that limit the UC process and decrease the UC emission intensity, respectively. The antenna effect (*vide supra*) is well known and uses chromophores with a high molar extinction coefficient to improve the Ln^III^ emission intensity. This approach was used to boost the UC emission intensity of the NaYF_4_: 20%Yb^III^, 2%Er^III^@NaYF_4_: 20%Nd^III^ NP by decorating the surface with the chromophore F-SG [2]. The only example of this strategy for improving the UC emission intensity of molecular systems was reported by Hyppänen and co-workers [195]. The system proposed was straightforward, the anionic [Er(tta)_4_]^-−^ complex and the IR-806 dye as the counter ion. Although a faint emission in the green was observed, upon excitation at 808 nm, the UC emission is mixed up into broad bands that might be residual ligand emission from the IR-806 dye or even from the tta ligands. Although the detailed UC mechanism was not proved experimentally, the authors proposed that the IR-806 absorbs the excitation, and transfers it to the Er^III^ excited levels. The field of molecular Ln^III^ UC systems is still in the initial development phase, and the possibility to use low power lasers in the biological window to obtain emission in the visible is exciting.

### 4.6. Sensing of Chemical Species inside Biological Systems Using Visible Emitting Ln^III^

Multimodal systems capable of luminescing and sensing of biologically relevant species are attractive due to the possibility of unraveling cellular processes and track abnormalities in the cell that are indicative of diseases [196,197,198,199]. Due to the possibility of controlling the chemical and spectroscopic properties by tailoring the ligand structure, Ln^III^ luminescent complexes are capable of sensing different chemical species [200,201,202,203,204,205,206,207]. The sensing process in those systems is based on the energy transfer chromophore → Ln^III^ that changes as a function of a chemical species. Using this approach, the emission intensity changes by the Ln^III^, is correlated with the concentration of a particular chemical species. Vitamin C [208], Cu^II^ [209,210,211], sulfide [209,210,211], carbon monoxide [138], biothiols [35], Zn^II^ [36], peroxynitrite [37], singlet oxygen [212,213,214], hypochlorous acid [139,215,216,217], superoxide anions [140], and ATP [218] are some of the compounds that can be sensed by Ln^III^ luminescent complexes.

**Figure 20:**
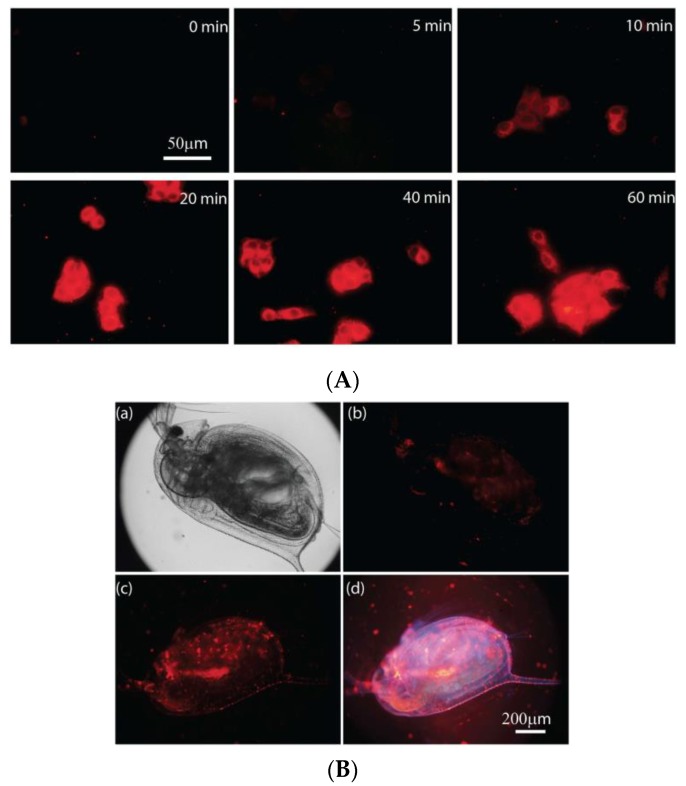
(**A**) Time-gated luminescence imaging of (**A**) HepG2 cells loaded with vitamin C, at different loading times, followed by incubation for 1 h with [Eu(tob)]^-−^ complex. [complex] = 20 μM, and [vitamin C] = 1.0 mM. (**B**) Luminescence imaging of *Daphnia magna* (**a**) bright field imaging, (**b**) time-gated luminescence imaging, (**c**) time-gated and (**d**) luminescence imaging after incubation with vitamin C for 40 min. [complex] = 5.0 μM, and [vitamin C] = 1.0 mM. Reproduced from [208].

Ascorbic acid (or vitamin C) is essential for healthy cell development, calcium absorption, and synthesis of collagen [219,220,221]. TEMPO-functionalized ligands were used to synthesize the complex [Eu(tob)]^−^, and sensing vitamin C in solution, in Hepg2 cells, and in *Daphnia magna* using time-gated luminescence (Figure 20) [208]. The presence of the TEMPO moiety quenches the Eu^III^ luminescence through the PET mechanism. In the presence of vitamin C, the TEMPO radical is quenched, resulting in the quench of the PET that reflects in the Eu^III^ emission intensity increase [208].

**Figure 21:**
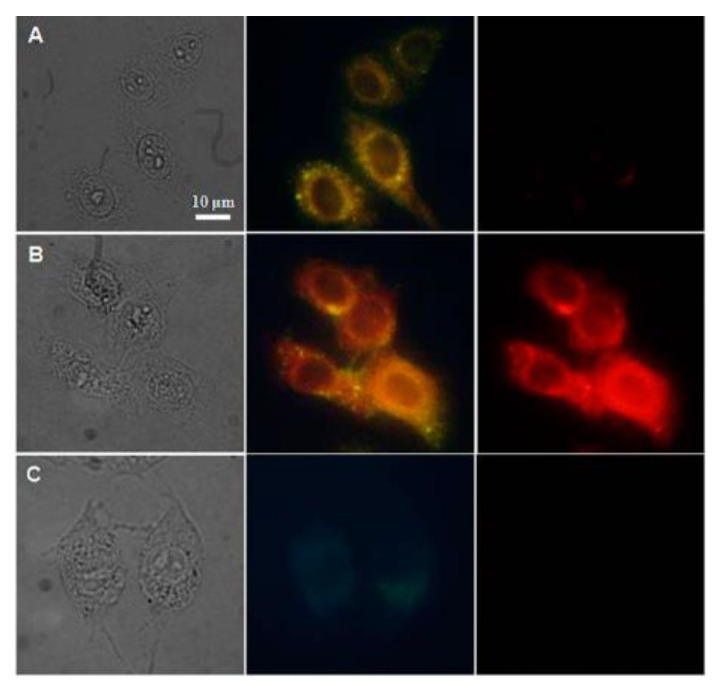
Luminescence imaging of HepG2 cells (**A**) incubated with [Eu(pfdap)_3_(tpy)] complex for 1 h, (**B**) incubated with ALA for 0.5 h followed by the [Eu(pfdap)_3_(tpy)] complex for 1 h, and (**C**) incubated with ALA for 0.5 h followed by the [Eu(pfdap)_3_(tpy)] complex for 1 h in the presence of NaN_3_. In all cases, the first, second, and third columns correspond to the bright field, luminescence imaging, and overlay of the bright field and luminescence imaging. After incubation with the [Eu(pfdap)_3_(tpy)] complex, the cells were irradiated at 660 nm for 0.5 to stimulate the production of singlet oxygen. ALA stimulates the production of singlet oxygen while NaN_3_ quenches it. [complex] = 20 μM, [ALA] = 15 μM, and [NaN_3_] = 200 μM. Reproduced from [212]. Copyright (2015) American Chemical Society.

Reactive oxygen species (ROS), are oxygenated compounds having unpaired electrons. ROS are generated during cellular processes, or by the interaction of the ligand excited state and the molecular oxygen [222,223,224,225]. For example, hypochlorous acid (HClO), a ROS produced by living cells, plays an essential role in immune systems. Accumulation of HClO triggers cell death and is associated with cardiovascular diseases, neurodegenerative disorders, and certain cancers [226,227,228,229,230,231]. Sensing of intracellular HClO using Eu^III^ complexes is achieved using ligands that are capable of reacting with HClO, and as a response, there is an increase or decrease in the emission intensity due to the changes in the energy transfer rates [139,215]. The sensing of HOCl inside RAW264.7 cells and *Daphnia magna* microorganisms using luminescence imaging was possible using the [Eu(npptta)]^-−^ complex that has a terpyridine-based ligand modified with a dinitrophenyl moiety [215]. In this system, there is quenching by PET due to the nitrophenyl moiety; in the presence of HOCl, the C=N bond is broken, resulting in an increase in the emission intensity due to the absence of PET. Singlet oxygen (^1^O_2_), another ROS, is produced by the interaction of triplet levels of the ligand with molecular oxygen, and is used in photodynamic therapy (PDT) [232,233,234,235]. The [Eu(pfdap)(tpy)] complex containing β-diketonate ligands functionalized with anthracene moieties was shown to be capable of sensing ^1^O_2_ inside Hepg2 cells using luminescence imaging (Figure 21) [212]. The Eu^III^ emission intensity increases as a function of the ^1^O_2_ concentration in the range between 5.0–1800 μM, and is specific to ^1^O_2_. The formation of the endoperoxide changes the energy transfer rate ligand → Eu^III^ making it possible to sense ^1^O_2_. Although not fully explained in the original research, it is possible that the triplet energy level of the pfdap ligand has similar or lower energy level than the excited Eu^III 5^D_0_ electronic level, reflecting in inefficient Eu^III^ sensitization. In the presence of ^1^O_2_, the formation of the endoperoxide decreases the electronic conjugation of the ligand. That reflects in an increase of the pfdap ligand triplet energy and, thus, a better sensitization of the Eu^III^ emission. In a follow-up study, the same research group used the [Eu(pfdap)_3_(dpbt)] complex that has excitation band red-shifted to 450 nm, a region that is more suitable for luminescence imaging [214]. The [Eu(pfdap)_3_(dpbt)] complex is capable of sensing ^1^O_2_ in MCF-7 cells, and in small microorganisms such as *Daphnia magna*.

Cu^II^ plays a central role in enzyme-catalyzed and redox reactions. High cellular levels of Cu^II^ are related to lethargy, increased blood pressure, liver damage, and neurodegenerative diseases [236,237]. Coordination of Cu^II^ using the dipicoylamine moiety in the ligand structure of the [Eu(bhhct-bped)] complex results in quenching of the Eu^III^ emission intensity, and allows sensing of Cu^II^ in Hepg2 cells [210]. This probe can be restored by “washing out” Cu^II^ using sulfide ions (S^2-^). An improvement of this probe was reported using a heterobimetallic Eu^III^/Tb^III^ complex containing a terpyridine-derivatized ligand (datp) that uses the intensity ratio between the ^5^D_0_ → ^7^F_2_ (Eu^III^) and ^5^D_4_ → ^7^F_5_ (Tb^III^) transitions for sensing [211]. Thus, the response of the probe does not change as a function of the instrumental response, setup, or concentration of the complex. Zn^II^ is involved in several extra- and intracellular processes; thus, its detection is relevant to unravel cellular processes [238,239,240]. Grafting chromophores and the [Nd(dota)] complex on zinc fingers yielded systems that are capable of sensing Zn^II^ [36]. In this exquisite system chromophore-zinc finger-[Nd(dota)], the distance chromophore → Nd^III^ changes as a result of the structural changes in the zinc finger structure caused by the presence of Zn^II^.

Adenosine triphosphate (ATP) serves as the chemical energy source for biological processes, including muscle contraction and maintenance of neuronal membrane potential [241]. The release of ATP to the extracellular space has been identified in both damaged and apoptotic cells [242,243]. Due to the strong interaction between the ATP phosphate groups and the Eu^III^, it is possible to sense in real-time changes in the intracellular concentration of ATP using the [Eu(dota^3^)(H_2_O)]^+^ complex inside NIH-3T3 [218]. The ATP molecules replace the coordinated solvent molecules reflecting in an increase of the Eu^III^ emission intensity that is a function of the ATP concentration.

## 5. Closing Remarks and Perspectives

The recent literature on Ln^III^ luminescent complexes shows a wide variety of possible applications in the fields of luminescence imaging and sensing of chemical species to aid in the understanding of biological processes or the diagnosis of diseases. The possibility to tailor the ligands’ structure to tune their chemical, spectroscopic properties, and develop multi-modal systems makes Ln^III^ luminescent complexes particularly attractive. Due to its high emission intensities, high emission quantum yields, and long luminescence lifetimes, Eu^III^ is the most used lanthanide in the synthesis of complexes for luminescence imaging, however, there is a high demand for the development of luminescent complexes that can be used in NIR-to-NIR imaging due to the high penetrability and low scattering of this light. Overcoming the high non-radiative rates, characteristic of NIR emitters, is still the big challenge for developing this field.

The field of molecular upconversion (UC) is expected to have fast development in the coming years. UC excitation is a cumulative effect of multiple first-order absorption phenomena where there is excited-state absorption (ESA) and energy transfer upconversion (ETU). In the UC process, a very long-lived intermediate state is present as opposed to the 2PA one, where a short-lived intermediate state is present. This results in a higher absorption cross-section in UC, making it possible to observe this process with inexpensive and low power continuous-wave lasers. The pair Yb^III^/Er^III^ is the most used system in UC due to the energy match that allows efficient ETU, the possibility of using NIR excitation, and emission in the blue, green, and red regions of the electromagnetic spectrum. To the date, only a few examples of molecular UC Ln^III^ complexes [189,190,191,192,193,194,195,244].

## Figures and Tables

**Table 1:** Summary of advantages and disadvantages of WF and CF microscopy.

Technique	Advantages	Disadvantages
WF microscopy	Wide range of excitation wavelengths, low cost	Does not allow the construction of 3D images, usually low signal-to-noise ratio
CF microscopy	Allows the construction of 3D images, high signal-to-noise ratio	The excitation wavelengths are restricted to specific wavelengths, high cost

**Table 2:** Formula, symmetry operation and selection rules of the magnetic dipole, electric quadrupole and electric dipole transitions [27].

Operator	Formula	Symmetry Operation	Selection Rules		
			*ΔS*	*ΔL*	*ΔJ*
Magnetic dipole ($\vec{M}$)	$-\frac{e\cdot h}{4\cdot \pi \cdot m\cdot c}\sum_{i=1}^{n}\left(\vec{l}+2\vec{s_{i}}\right)$	Rotation (*R_x_, R_y_* and *R_z_*)	0	0	0, ±1
Electric quadrupole ($\vec{Q}$)	$\frac{1}{2}\sum_{i=1}^{n}\left(\vec{k}\cdot \vec{r_{i}}\right)\cdot \vec{r_{i}}$	Product (*xy*, *xz*, *yz*, *x^2^ − y^2^*)	0	0, ±1, ±2	0, ±1, ±2
Electric dipole ($\vec{P}$)	$-e\sum_{i=1}^{n}\vec{r_{i}}$	(*x*, *y* and *z*)	0	≤ 6	≤ 6 (2, 4, 6)

**Table 3:** Cell line names and abbreviations.

Cell Line	Abbreviation	Cell Line	Abbreviation
Human liver carcinoma	Hepg2	Glioblastoma	NG97
Human hepatic cells	L02	Human pancreatic cancer	PANC1
Mouse skin fibroblast	NIH-3T3		
Chinese hamster ovarian	CHO	Cervical cancer	HeLa
Non-small human lung carcinoma	H460	Abelson murine leukemia virus-induced tumor	RAW 264.7
Mouse fibroblast	L929		
